# Evaluating Patient Usability of an Image-Based Mobile Health Platform for Postoperative Wound Monitoring

**DOI:** 10.2196/mhealth.6023

**Published:** 2016-09-28

**Authors:** Rebecca Gunter, Sara Fernandes-Taylor, Andrea Mahnke, Lola Awoyinka, Chad Schroeder, Jason Wiseman, Sarah Sullivan, Kyla Bennett, Caprice Greenberg, K Craig Kent

**Affiliations:** ^1^ Wisconsin Institute of Surgical Outcomes Research University of Wisconsin Madison, WI United States; ^2^ Marshfield Clinic Research Foundation Biomedical Informatics Research Center Marshfield Clinic Marshfield, WI United States; ^3^ Department of Surgery University of Wisconsin Madison, WI United States

**Keywords:** telemedicine, smartphone, surgical site infection, postoperative wound infection

## Abstract

**Background:**

Surgical patients are increasingly using mobile health (mHealth) platforms to monitor recovery and communicate with their providers in the postdischarge period. Despite widespread enthusiasm for mHealth, few studies evaluate the usability or user experience of these platforms.

**Objective:**

Our objectives were to (1) develop a novel image-based smartphone app for postdischarge surgical wound monitoring, and (2) rigorously user test it with a representative population of vascular and general surgery patients.

**Methods:**

A total of 9 vascular and general surgery inpatients undertook usability testing of an internally developed smartphone app that allows patients to take digital images of their wound and answer a survey about their recovery. We followed the International Organization for Standardization (ISO) 9241-11 guidelines, focusing on effectiveness, efficiency, and user satisfaction. An accompanying training module was developed by applying tenets of adult learning. Sessions were audio-recorded, and the smartphone screen was mirrored onto a study computer. Digital image quality was evaluated by a physician panel to determine usefulness for clinical decision making.

**Results:**

The mean length of time spent was 4.7 (2.1-12.8) minutes on the training session and 5.0 (1.4-16.6) minutes on app completion. 55.5% (5/9) of patients were able to complete the app independently with the most difficulty experienced in taking digital images of surgical wounds. Novice patients who were older, obese, or had groin wounds had the most difficulty. 81.8% of images were sufficient for diagnostic purposes. User satisfaction was high, with an average usability score of 83.3 out of 100.

**Conclusion:**

Surgical patients can learn to use a smartphone app for postoperative wound monitoring with high user satisfaction. We identified design features and training approaches that can facilitate ease of use. This protocol illustrates an important, often overlooked, aspect of mHealth development to improve surgical care.

## Introduction

Telemedicine has begun to supplement, and in some cases supplant, postoperative care received in the clinic in many surgical practices. Existing platforms include Web and mobile phone–based portals for virtual follow-up after elective general surgery and telephone follow-up after laparoscopic cholecystectomy and open inguinal hernia repair [1-3]. These platforms have been met with wide acceptance and enthusiasm by patients and their surgeons in the low-risk, elective surgery cohorts studied [4]. However, patients have not been rigorously included in the design of these apps despite an extensive literature on user-centered design in the scientific literature from the disciplines of medical informatics and human-computer interaction [5-8]. Indeed, recognizing the importance of involving users in the development of new devices and protocols, the Food and Drug Administration (FDA) has mandated consideration of the user experience in their Quality System Regulation [9].

As ownership of tablets and mobile phones becomes more common [10], patients and their caregivers are increasingly willing to use technology to access care [11]. Alongside this trend, policy mandates have made improving transitions of care following hospital discharge and reducing hospital readmissions a national priority [12-15]. These trends together create an enormous opportunity for telemedicine to improve transitions of care for surgical patients. However, with increasing enthusiasm for telemedicine, new platforms must be rigorously vetted by patients, the end users, to ensure their full acceptability and accessibility. This can be achieved through the use of established user-centered design guidelines, which comprise a diverse set of concepts and methods grounded in human factors engineering and ergonomics that facilitate the usability of technology for the target user. Although clinical outcomes from the studies of existing telemedicine platforms in surgical practice are encouraging, they are limited by substantial bias—more than 80% of published telemedicine interventions include only those patients who can access or are familiar with the necessary technology (eg, tablet or mobile phone), resulting in the exclusion of between 12% and 56% of otherwise eligible participants [16]. Additionally, much of the prior research of telemedicine protocols for surgical patients have focused on routine procedures that already have a low base rate of postoperative and postdischarge complications [1,2,17]. As a result, major knowledge gaps remain regarding whether telemedicine can be used to monitor a higher-risk population that is less familiar with mobile technology and what is required of novice technology users to successfully complete such protocols.

In addition, many existing telemedicine platforms designed for the postdischarge period are primarily text or audio based but transmit no visual information [2,3,18]. A crucial component of postoperative and postdischarge recovery is appropriate healing of the surgical wound. The addition of a visual component (video and images) allows more complete evaluation of wound healing, which is vital for monitoring postoperative recovery for 3 primary reasons: wound infection is the most common nosocomial infection in surgical patients, it is a leading cause of hospital readmission [19] as infections increasingly develop after hospital discharge [20], and patients are unable to identify wound complications with a high rate of false negatives [21,22]. Telemedicine protocols that rely on mobile devices, collectively termed mobile health (mHealth), are uniquely positioned to easily provide visual information, essential to the diagnosis of a wound infection.

Those telemedicine protocols that do have a visual component are frequently asynchronous and episodic and have not been designed for ongoing monitoring of postoperative recovery. Most commonly, these protocols involve either digital images or videoconferencing intended to replace an in-person office visit [1,17,23-25]. However, while these are useful in their ability to decrease travel time and cost, they are not sufficient in diagnosing an early wound complication for reasons stated above, namely that, a surgical site infection (SSI) often develops before many follow-up visits are scheduled. Other protocols intended for wound monitoring, such as the mobile Post-Operative Wound Evaluator (mPOWER), are intended to allow patients to submit images, but do not guarantee that a provider will review them unless notified to do so [26,27]. Unless patients alert their provider regarding a concerning finding, something patients are not reliably able to do, such protocols may inadequately detect the early signs of a wound complication.

We address these gaps by creating an image-based smartphone app aimed at increasing communication between patients and their caregivers after they leave the hospital as part of a forthcoming effort to detect wound complications at an early stage and to reduce hospital readmissions. We then evaluate its usability in a largely technology-naive population of patients undergoing general and vascular surgery. In constructing this project, we consulted 2 international standards: International Organization for Standardization (ISO) standard 9241-12 was used to optimize the design of our application and then ISO 9241-11 was used to guide usability testing of the app. ISO 9241-11, a widely used guideline for current usability testing methods, which focuses on effectiveness (ie, task completion), efficiency (ie, time within task), and user satisfaction of new technology, was used to assess the patient-centeredness and usability of this app to monitor postoperative wounds [28]. To our knowledge, we are the first to invoke ISO 9241-11 to assess an image-based app in a clinical patient population. Our findings have the potential to provide vast amounts of clinically vital information that has been otherwise unavailable to health care providers. We also address the utility of existing usability standards for image-capturing mHealth platforms.

## Methods

### Subjects

Eligible participants included inpatients 18 years of age or older on the vascular or general surgery service of a large, academic tertiary care hospital. Subjects were recruited during one of two usability sessions in November and December 2015. Participants were eligible if they had a surgical incision longer than 3 cm and were close to their baseline functional status.

**Table 1:** User interface design dimensions from International Organization for Standardization (ISO) standard 9241-12 and corresponding WoundCheck design features.

Information display dimension	Definition	Method employed	Sample app design features
Clarity	Content conveyed quickly and accurately	Physician review; focus group	All app language validated by physician review panel (for clinical usefulness) and lay focus group (for interpretation)
Discriminability	Information is readily distinguished	Iterative redesign	Tap-only response options (no text entry or scrolls) Consistent 3D button placement below text Redesign of image capture screen to prevent errors and reduce wrong-button taps Color-coded buttons
Conciseness	No extraneous content	Focus group review of content	Yes or no questions for symptoms Draft language reviewed by focus group to reduce word count while retaining interpretation
Consistency	Information is presented in the same way consistent with expectations	Focus group review of layout; Iterative redesign	All response screens are identical Each module contains review screen prior to submission
Detectability	Attention is directed to salient information	Multidisciplinary design team; physician review	Image review screens to ensure quality image Feedback screens to track and confirm submission Buttons are 100x100 pixels (0.33 in) or larger Font fills the frame
Legibility	Easy to read content	Focus group test; iterative redesign	Readable Helvetica Neue bold font choice, size 26 or larger with high contrast display (black type on white background) Button shadowing
Comprehensibility	Meaning is unambiguous and clear	Focus group review of content; physician review	6th grade reading level Focus group read-back of app questions “in your own words” to ensure faithful interpretation Focus group feedback for image capture training

 Subjects with major cognitive or neurologic deficits prohibiting their independent use of the app were included only if they had a capable caregiver who consented to complete the app on their behalf. All subjects who met inclusion criteria were approached to participate. Participants were asked regarding their prior experience with smartphones, whether they owned their own smartphone, and whether they had used a smartphone to take a digital image.

We aimed for a sample size of at least 5 participants, a number based on evidence from the usability literature indicating that 5 participants make a sufficient sample size to detect 80-85% of an interface’s usability problems [29]. We continued to enroll purposively past our sample size goal to utilize the remaining time.

The University of Wisconsin Health Sciences Institutional Review Board approved the study protocol.

### The App

WoundCheck is an iOS app that enables patients to capture digital images of surgical wounds and sends them to their providers from home, along with brief updates on postoperative recovery. This app was developed internally through the University of Wisconsin Department of Surgery with the assistance of software programmers in our Information Technology division. In designing the app, we consulted ISO 9241-12, an international standard for screen layout and the visual display of complex information, and established guidelines on user interface design to ensure that the user interface was easily navigated by our target population of older adults and novice users [30,31]. Table 1 summarizes the app’s features and the method of development vis-à-vis the salient dimensions of the ISO standard for user centered design including clarity of the content, discriminability of information, conciseness, consistency of presentation, detectability, legibility, and comprehensibility. The app is accompanied by a training program to be delivered prior to discharge that draws on evidence-based tenets of adult learning and memory retention (Table 2), in keeping with similar transitional care programs targeting older adults [32-34]. Among these tenets is the need for adult learners to feel actively engaged in the learning process, to frequently receive positive reinforcement, and to set the pace of learning. We allowed ample time for questions and for participants to interject comments. We also allowed participants to use the smartphone and the app directly after a short demonstration, engaging visual, auditory, and kinetic forms of learning. Adult learners also require repeated exposure to new material and to have it presented in a variety of formats. Each participant received a training booklet that reinforced the steps of the app for reference if questions arose after discharge.

**Table 2:** Tenets of adult learning and memory and corresponding training design features.

Evidence-based dimension of adult learning	Sample training design features
Require more time to learn new skills [35]	Let participant set the pace of training
Need repetition and multiple formats of materials [36]	Repetition; supplementary flash cards; let participant develop own narrative around the device
Challenged by complex, unusual material [37,38]	Emphasis on purpose of training; emphasize “why” of tasks
Decline in motivation when not experiencing success [38]	Frequent positive feedback; opportunities to reflect and ask questions throughout
Repeated exposure facilitates learning [39,40]	Primary training session + refresher training prior to discharge
Cue-based recall [41]	Use of reminder alarm at the time of participant choosing as a cue to use app
Task performance (not just observation) with teach-back [41]	Provide a device to participant to use throughout training

 

**Figure 1:**
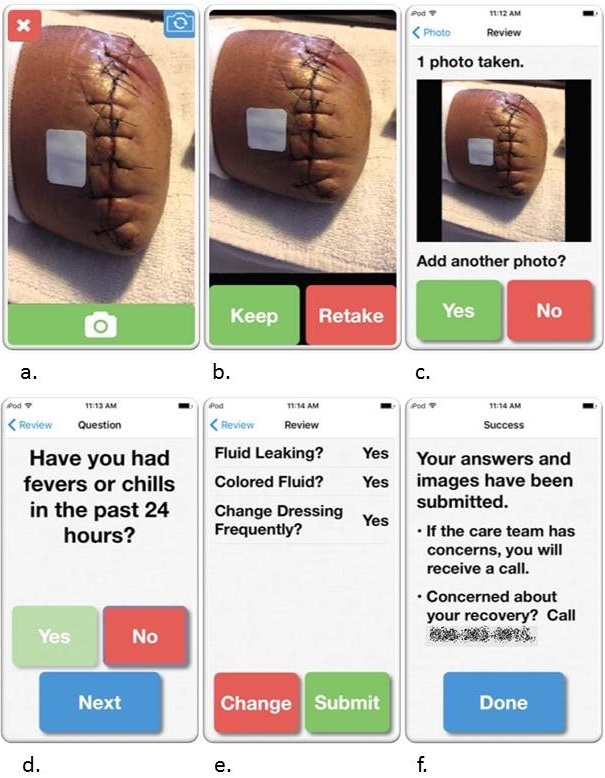
Screenshots of the final app. A. Modified camera screen. B. Image review screen where participants can choose whether to keep the image they have taken or try again. C. Review screen of all added images; up to 4 images may be added. D. A series of yes or no questions follow. E. Participants can review their survey responses and have the option to change them prior to submission. F. Submission confirmation screen.

The program is ultimately designed for use during the period between hospital discharge and the routine postoperative clinic visit. The app was designed to be linear with one pathway through the app to maintain simplicity and intuitiveness. There are 2 phases to the app: an image-taking phase where participants take digital images of their wound and have the ability to review or retake their images, and a brief survey with yes or no questions regarding their recovery. Screenshots of the app are provided in Figure 1, and survey questions are provided in Textbox 1.

To vet the content of the app and training and meet the burden of the ISO design standard, we conducted 2 focus groups to review the app with Community Advisors on Research Design and Strategies (CARDS). These are standing focus groups of community members from diverse racial, ethnic, socioeconomic, and educational backgrounds who are recruited from food pantries, senior meals, parenting programs, and other similar programs. They are trained to give constructive feedback to researchers, health educators, and outreach professionals. The CARDS members, the majority of whom are novice smartphone users, evaluated prototype screens of the app and all app language in the first focus group. The image capture training protocol was evaluated in the second focus group.

### Health Insurance Portability and Accountability Act Compliance

**Figure 2:**
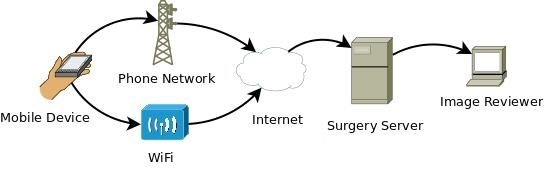
Wound Check app data flow overview.

The app and transmission of patient data were developed to fully comply with the Health Insurance Portability and Accountability Act. A passcode is used to secure and encrypt the device. Each device is profiled, allowing us to remotely wipe the device, prevent the installation of additional apps, and limit other device features. No information is stored on the mobile phone itself; the app can only be used to submit information, not retrieve it. The app transmits data to the University of Wisconsin Department of Surgery research server using the Hypertext Transfer Protocol Secure (HTTPS; Figure 2). A unique nonmedical record number identifier is used for each participant. No identifying information is transmitted, and participants were instructed not to send pictures that included identifying marks or their face. If the participant is idle for more than 10 minutes during data collection, the app times out and the data is deleted. Only research personnel with responsibility to review images have access to the submitted images. The system automatically logs off users after 30 minutes of inactivity. Audit controls monitor access.

**Questions included in the survey portion of the WoundCheck app.**

1. Have you have fevers or chills in the past 24 hours?

2. Have you changed how you take your medication in the last 24 hours?

   2a. (If responded yes to 2) Is this change related to your pain medication?

   2b. (If responded yes to 2a) Did you increase your pain medicine?

3. Has the area around your wound become red in the past 24 hours?

4. Has the area around your wound become swollen in the past 24 hours?

5. Is there a bad smell coming from your wound?

6. Is fluid leaking from your wound?

   6a. (If responded yes to 6) Is the fluid white, yellow, or green?

   6b. (If responded yes to 6) Do you change the dressing more than once because fluid soaks through?

 

### User Tasks

Following preliminary design, we formally tested the usability of the app with postoperative vascular and general surgery patients at a major academic medical center. The app was loaded onto a 5^th^ generation iPod Touch running iOS8. We assessed patients’ baseline familiarity with smartphones prior to testing. A researcher introduced the device to participants with an overview of its general functions and how to operate it, if needed. User tasks included waking up the device, launching the app, image capture, review and retake or acceptance of captured images, question response, and submission. Following the first round of usability testing, an interim assessment of the app was performed and adjustments were made based upon the findings of the first round. The updated version of the app was then used for the second round of testing.

### Measures and Analysis

We consulted ISO 9241-11 in designing the format for formal usability testing of the app [28]. Effectiveness (ie, the ability to successfully complete each task independently and whether assistance was required) and efficiency (ie, the time needed to complete each task) were measured by direct observation and by mirroring of the device onto a research computer using the software AirServer (App Dynamic). The mirrored screen on the laptop was recorded using Morae (TechSmith) screen recording software. Training sessions were audio recorded for later review.

Following usability testing of the app, participants were asked to rate their performance and to provide feedback on the app itself. Participants also completed a system usability scale (SUS) to evaluate their satisfaction with the app (questions presented in Textbox 2) [42,43]. Images generated during the testing sessions were independently reviewed by 3 physicians to assess whether they could be used for diagnostic and treatment purposes. If a reviewer deemed an image as not usable, they were asked to provide a reason.

**System usability scale questions. Responses followed a 5-point Likert scale from “strongly agree” to “strongly disagree.”**

**Statement**

- I think that I would like to use this app frequently
- I found the app unnecessarily complex
- I thought the app was easy to use
- I think that I would need the support of a technical person to be able to use this app
- I found the various functions of this app were well integrated
- I thought there was too much inconsistency in this app
- I would imagine that most people would learn to use this app very quickly
- I found the app very awkward to use
- I felt very confident using the app
- I needed to learn a lot of things before I could get going with this app

## Results

### Participant Characteristics

Of the 14 patients who were approached to participate, 5 declined due to time constraints or disinterest. Nine participants completed usability testing, 3 of whom had caregiver assistance or proxy participation. Five participants owned their own smartphone, and 7 had used a smartphone to take a digital image at least once prior to this study, leaving 2 who had no prior experience with smartphones. Demographics and basic clinical information are presented in Table 3.

Four participants (44.4%) had abdominal wounds (an aortic graft explantation and an axillary-bifemoral bypass, 1; an aortobifemoral bypass, 1; an open distal gastrectomy, 1; and an open distal pancreatectomy and splenectomy, 1). Four participants (44.4%) had groin wounds (an aortobifemoral bypass, 1; bilateral groin explorations and repair of a common femoral artery aneurysm, 1; a superficial femoral artery graft resection and interposition graft placement, 1; and an endovascular aortic aneurysm repair, 1). Two participants (22.2%) had lower extremity wounds (bilateral lower extremity fasciotomies, 1; and a superficial femoral artery to posterior tibial artery bypass, 1). One participant (11.1%) had an amputation stump above the knee. Two participants had 2 wounds, bringing the total number of wounds to 11.

**Table 3:** Demographic and baseline characteristics.

Characteristic	n (%) or mean (SD)
Female, n (%)	5 (55.6)
Age (years), mean (range)	55.2 (19 - 80)
**Race, n (%)**	
White	6 (66.7)
African-American	2 (22.2)
Latino	1 (11.1)
Body mass index (kg/m^2^), mean (range)	29.0 (17.4 - 43.65)
**Insurance status, n (%)**	
Private	4 (44.4)
Medicare	3 (33.3)
Medicaid	1 (11.1)
Uninsured	1 (11.1)
**Incision site, n (%)**	
Abdominal	4 (44.4)
Groin	4 (44.4)
Lower extremity	2 (22.2)
Amputation stump	1 (11.1)

**Table 4:** Effectiveness, efficiency, and satisfaction results of usability testing.

Participant	Training time (min)	Time to complete app independently (min)	Total time (min)	Required assistance?	Image deemed usable by majority of raters	SUS^a^ score
**Session 1**							
P1	12.8	1.6	14.4	No	Yes (AKA^b^ stump)	82.5
P2	2.7	3.1	5.8	No	Yes (Abdomen)	97.5
					Yes (Groin)	
P3	6.4	16.6	23.0	Yes	No (Groin)	72.5
P4	2.2	2.4	4.6	No	Yes (Abdomen)	87.5
Session 1 mean (SD)	6.0 (4.9)	5.9 (7.1)	12.0 (8.6)			85 (10.4)
**Session 2**							
P5	2.4	1.4	3.9	No	Yes (BLE^c^ fasciotomies)	82.5
P6	3.2	6.2	9.4	Yes	Yes (Groin)	87.5
P7	2.1	6.4	8.5	Yes	Yes (Lower extremity)	75
					No (Groin)	
P8	8.0	4.7	12.7	Yes	Yes (Abdomen)	70
P9	2.9	2.2	5.1	No	Yes (Abdomen)	95
Session 2 mean (SD)	3.7 (2.4)	4.2 (2.3)	7.9 (3.5)			82 (9.9)
Overall							
mean (SD)	4.7 (3.7)	5.0 (4.7)	9.7 (6.2)			83.3 (9.6)

^a^SUS: System Usability Scale (scored 0-100).

^b^AKA: above the knee amputation.

^c^BLE: bilateral lower extremity.

### Effectiveness and Efficiency

Effectiveness and efficiency data are presented in Table 4. The mean length of time spent with each participant for the full app training session, excluding study introduction and survey completion, was 9.7 minutes (range: 3.9-23.0 minutes). The mean length of time participants needed to complete the app independently was 5.0 minutes (range: 1.4-16.6 minutes). For all of these measures, the participants in the second round (ie, users of the updated version of the app) had better efficiency over the participants in the first round (ie, users of the app in its original form). Forty-four percent of participants needed prompting or assistance from a member of the research team to complete the app; 55.6% were able to complete the app in its entirety without assistance. Of the documented instances when researcher’s assistance was given, 64% were related to taking images of wounds, most often related to participant positioning and navigating the device’s camera functionality.

**Figure 3:**
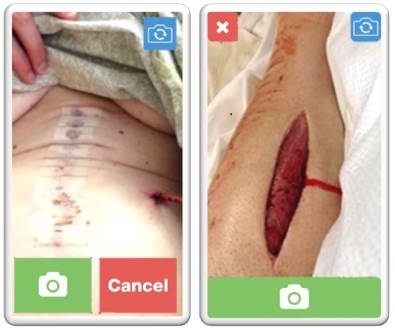
Original and modified image-taking screen. On the left is the original camera screen with both the image-capture and cancel buttons at the bottom of the screen. On the right is the modified screen based on user feedback. The image-capture button takes up the whole bottom of the screen, but does not extend as far up into the screen, and the cancel button has been moved away from it to decrease button confusion.

The most difficult task in the initial round of testing was to take a digital image of the wound. Participants were confused about the flow through the image-taking portion of the app, and they also faced difficulty with button placement. Specifically, the placement of the image capture button directly next to the cancel button led to image capture attempts that resulted in cancellation. In addition, the cancel button looped back to restart the app rather than sending participants forward even if they had already captured an image. As a result of these difficulties, the image-taking portion of the app was redesigned to make it more intuitive, and the camera buttons were placed in more convenient locations on the screen to facilitate image capture (Figure 3). Following these adjustments, participants in the second round of testing had less difficulty with this section. Novice smartphone users also experienced confusion with changing the direction of the camera to face toward or away from them and required frequent reminders and assistance.

Participants with groin wounds, and particularly obese participants with groin wounds, had considerable difficulty taking images of their wound independently due to inadequate exposure of the wound. At least one other person was required to fully expose the wound, and even then, it was difficult to achieve the optimal angle for image capture. Participants who had active caregivers present were better able to perform this task without requiring researcher’s assistance.

On assessment of image quality, 9 of 11 (81.8%) images were deemed sufficient for diagnostic purposes by a majority of rating physicians (Table 4). Five of 11 images had at least one physician rate it insufficient, primarily because the entirety of the wound was not visible in the image (scope). One of these was a patient who was too close to surgery to fully uncover and visualize her wounds. Another patient had the very top of his abdominal incision covered by his gown but otherwise had an adequate image. A man with an amputation stump generated an image that had insufficient lighting for one rater to comfortably say whether there was erythema or ecchymosis, which was a function both of how wound healing appears in darker skin and the available light. The 2 wounds that the majority of raters found inadequate for clinical use were 2 of the 3 groin wounds; this was consistent with the participants’ difficulty in taking the picture during usability testing, for the reasons stated above.

The survey task within the app was easy for all participants to use. On the initial round of testing, the screen for reviewing survey responses was scrollable, such that all responses appeared on a single screen, but some were not visible unless the participant scrolled to the bottom of the screen. This was confusing for some participants, as this was the only scrollable screen within the app, requiring mastery of a new functionality. The response review screen was revised in the second round of testing to be split into 2 screens to eliminate the need to scroll. After this adjustment, participants had no difficulty with this section.

### Usability

The responses to the System usability scale (range: 0-100) are presented in Table 4. The overall usability score for the app was 83.3, which is considered good for usability testing [44]. Most participants found the app easy to use, though the questions that did not elicit a unanimous positive response (“I think I would need the support of a technical person to be able to use this app,” “I would imagine that most people would learn to use this app very quickly,” and “I needed to learn a lot of things before I could get going with this app”) indicate a degree of tentativeness regarding participants’ ability to independently complete the app. One participant said she would “probably have to write the steps down” to be able to complete it independently, though said she “didn’t find it that complex once (she) got into it” and that she “would do it because we need to do it.” Another said she could imagine “a lot of people who would have all kinds of problems” learning to use the app.

These challenges were also observed during usability testing, particularly with novice users who, in addition to learning to use the app, needed more time to become comfortable using the device itself. Four participants struggled with simply tapping the screen and alternating between tapping icons on the screen and pressing the home button; two came close to deleting the app by pressing the icon for too long rather than tapping it. As stated previously, novice users also struggled with using the camera, particularly with switching the direction of the camera to face them.

The most commonly cited concerns regarding the protocol were confidentiality of patient information and whether anyone in the care team would actually review the submitted images and survey responses. One participant was concerned “whether information (would be) followed through,” saying “you might have taken lots of pictures, but if no one looks at it, it’s all for nothing.” Other concerns raised were device battery life and difficulty being able to fully visualize the wound to take a digital image. Three participants stated they had no concerns. All 9 participants said they would be able to complete the app daily after discharge if they were given full instructions. One particularly enthusiastic participant said, “I wish I had it today.” All nine said they would benefit from a protocol using this app following hospital discharge. One participant said, “I think it’s really pretty neat...if you have a concern, you’ll get an answer like that.” Eight participants said they would recommend the app to a friend or family member if they had surgery, and one participant was neutral, saying “...that’s their decision.”

## Discussion

### Principle Findings

The current standard of care for the majority of surgical patients following hospital discharge involves little formal communication between patients and their care team until their routine clinic follow-up 2-3 weeks after discharge. This is a crucial time period during which many complications and setbacks to recovery occur, and is thus ripe for mHealth innovation [45]. Other mHealth protocols have been developed to improve patient monitoring or replace routine postoperative clinic visits [1,3,27]. However, these protocols are limited in their episodic follow-up, the lack of guaranteed provider review, or the lack of any transmitted visual information.

To address these gaps, we have developed a smartphone app that allows patients to be in daily communication with their provider with both subjective symptom data and visual information in the form of digital images. We have demonstrated that most patients and their caregivers are able to learn to use our app, can use it to transmit meaningful clinical information, and have a high level of satisfaction and enthusiasm regarding the protocol. Additionally, studying patients during the immediate postoperative period allows for the most conservative estimate of usability given that patients are still in recovery and may not be at their functional baseline. Given that our participants were mostly older adults, seen during the vulnerable postoperative period, some with very limited prior smartphone experience, the wide success we observed is encouraging for the ability of the general population to use the app without difficulty once given protocol-based training and clinical support at the outset.

Insights from the field of systems engineering provide a helpful framework for the development of mHealth protocols, as well as their attendant training programs. Work focusing on universal access and assistive technology for persons with disabilities is especially relevant for creating mHealth protocols accessible to a diverse patient population, particularly patients recovering from surgery, who are elderly or have limited prior experience with the technology, as in this study. Vanderheiden [5], a systems engineer with a focus in user experience optimization, outlines 3 approaches to assist in those efforts that are as follows: changing the individual, providing adjunct tools, and changing the environment.

For the purposes of our protocol, changing the individual involved tailored training, which we made modular so that portions could be added or skipped depending on the participant’s needs. As expected, the participants who struggled the most with the app were novice smartphone users and older participants. Most of this difficulty was in learning to navigate the smartphone itself and not necessarily related to the app. This was reflected in the responses to the system usability scale, where 11-20% of participants expressed needing to learn a lot before they could get going with the app or felt that they would need assistance of a technical person to complete it. Previous studies of mHealth apps have found similar results, with lack of familiarity with mobile devices and the need for assistance identified by participants as barriers to independent use [46-48]. As a result of this added difficulty, novice users of smartphones required dedicated training to become facile using the device before moving on to training specific to the app; those participants who were familiar with the device were able to skip this portion of training. This flexibility in training was envisioned prior to usability testing, but by doing formal usability testing, we were better able to identify components of the protocol that needed dedicated training and for which patients they were needed.

Importantly, efficiency of training should not come at the expense of effectiveness. Protocol training will need to be performed at the pace of the learner, taking care to keep them engaged. Two participants expressed training fatigue, with one saying, “I’m glad you’re getting out of here; that was time consuming” after 27 minutes of training, despite her not having fully mastered the task. Another said, “you mean we’re not done?” after 25 minutes of training. Bearing this in mind, future training efforts may need to be spread over multiple sessions both to reinforce tasks and to avoid fatigue and boredom with a single session.

The second approach for improving accessibility is to provide adjunct tools to overcome particular barriers to use. For participants who struggled with tapping the screen, a stylus may be easier and more intuitive than using their finger. One participant opted to do this on her own based on her prior experience using a stylus with her tablet device. Another barrier we encountered in our protocol was the difficulty experienced by patients with wounds in certain locations that were difficult to take an image of, particularly groin and abdominal wounds as well as amputation stumps. Potential tools to aid these patients might include training them to use selfie-sticks or mirrors to improve their ability to independently take images of wounds in these locations. However, assistive devices or tools have the potential to add an additional layer of complexity for patients who are already uncomfortable with the device or the app, and this must be weighed against the potential benefit of their use. Because groin wounds are at increased risk of developing surgical site infection [49], these are the very patients who stand to gain the most from postdischarge wound surveillance, and every effort should be made to maximize their ability to participate, which may also include identifying a competent caregiver willing to assist.

Finally, user accessibility may be improved by changing the environment to be accessible to all users without the need for specialized devices or tailoring to the individual, an approach termed “universal design.” Following the first round of testing, we made several subtle but significant improvements to the design of the app itself to improve its usability for a wide range of users. The reconfiguration of buttons on the camera screen made capturing images easier for participants with limited fine motor ability or who had difficulty with discrete touch. We eliminated screens that required scrolling up and down to preclude novice users or those with cognitive limitations from having to learn an additional skill. In making these changes, the app becomes more accessible to all users, including those who did not have difficulty completing it prior to these modifications, by making it as simple and straightforward as possible. mHealth platforms in the future should strive for universal accessibility in their design to maximize participation and benefit.

One aspect of universal design we did not achieve was making the app compatible with an Android device. For those participants more familiar with Android technology than iOS, learning to use the app first required learning to use the device, a barrier not experienced by those participants who had used an iOS device in the past. This is particularly important given key demographic differences in smartphone ownerships, specifically that minorities, those of lower income, and those with lower educational attainment are more likely to own an Android device [50]. Future iterations of this app should be made Android-compatible to increase its usability for a wider range of patients.

However, despite our best efforts to incorporate these insights from systems engineering and develop a universal design for the app and for our training protocol, it is likely that some patients will still need the assistance of a caregiver to complete the app. Through usability testing, we identified several possible reasons why some might be unable to complete the app independently. Those patients who are novice smartphone users and are unable to learn to complete the app independently will by definition need assistance. Patients who have wounds in locations they cannot reach or cannot visualize sufficiently on their own will need a caregiver. Additionally, patients who have limited independence at baseline will need assistance, as with one of our participants who was a hemiparetic bilateral lower extremity amputee. In these cases, a competent caregiver or family member will need to be identified so that these patients may still benefit from mHealth protocols. These patients may already have a caregiver or involved family member due to their baseline functional status and reliance on others for aspects of their care.

Interestingly, participants consistently rated themselves as having successfully completed the app, even when their performance did not warrant such an assessment. When asked whether taking a digital image of their wound was easy to complete, only 2 participants were neutral, while all others agreed or strongly agreed. All 9 participants agreed or strongly agreed with the statement “I am confident I completed this task” in reference to taking a digital image of their wound, even the participants whose images were not sufficient for clinical decision making. Sonderegger et al [51] found a similar trend in their study of mobile phone usability in older adults. They posited several possible explanations for this finding. One was that this may have been a result of low expectations participants had for themselves, such that they overstated even small successes. Another was that participants may have felt that with practice they would eventually be successful, valuing their potential success over their actual success. This is an important finding, indicating that participants using new technology need to be carefully educated about what is expected of them and what constitutes meaningful success.

Despite these barriers, there was substantial enthusiasm from most participants about the protocol. One participant told the research team he wished he could take the device home upon discharge and use it to stay in contact with the care team. All participants thought they would benefit from this protocol and would be willing to complete the app daily if they were instructed to do so. This is consistent with previous studies of mHealth [11,26,52,53], which collectively indicate that patients and their caregivers are willing to participate in a variety of remote monitoring protocols, see such protocols as being potentially beneficial to them, and are satisfied when they participate.

In addition, the fact that many participants could ultimately complete the app independently or with caregiver’s assistance is encouraging. The overall usability score of 83.3 is above average for usability testing, indicating a level of comfort among first-time users of the app [54]. Following a short training session, most patients will be able to participate in a protocol using this app, though as stated above, certain populations will likely need more focused training.

This is the first study, to our knowledge, to formally investigate usability of a medical device with digital image taking capability using the ISO 9241-11 standards [28]. Our findings indicate that patients are capable of completing such an app and that there is broad enthusiasm for its use. However, increased attention will need to be paid to novice users and older adults who may need more extensive training before they will be able to complete mHealth protocols independently. Additionally, to avoid widening of existing disparities in access and health outcomes, health systems must ensure such protocols, if proven beneficial, are available to all patients and not only to those who already have access to the necessary technology. As health systems increasingly focus on improving transitions of care and maximizing outpatient management of complex patients, the ability to monitor recovery of conditions that have a physical manifestation, including fields beyond vascular and general surgery, this app and those similar to it have the potential to revolutionize the way care is delivered in the postdischarge period.

The results of this study should be interpreted in the context of several limitations. Our study may be limited by its sample size. Considerable debate exists within the literature regarding the ideal sample size for usability testing. Historically, a sample of only 5 participants was thought to be of sufficient size, but more recent data suggests a larger sample is required to make accurate assessments [29,55-57]. However, the more recent estimates for ideal sample size were based on usability testing of more complex websites with multiple possible pathways. Given the simplicity and linearity of the app in this study and the diversity of the participants studied, we feel confident that all major areas for improvement within the app were identified and addressed in the redesign of the app. In addition, our results may be limited by the fact that data was collected only at one medical center; our findings may be specific to our patient population and need additional testing in other patient populations with different sociodemographic or cultural characteristics. Moreover, while the training was performed by a researcher for the purposes of this study, it is likely that this would need to be performed by a nurse in the clinical setting. Further work will need to be done to examine implementation and feasibility of this protocol outside of a controlled research setting.

### Conclusion

As postoperative lengths of stay decrease, health systems will need to become creative in their methods of monitoring patients in the outpatient setting. Many telemedicine protocols have emerged to address this goal, but ours is the first to add an asynchronous visual component through the use of digital images, whose power to efficiently convey vast amounts of information is unparalleled in today’s standard of care. Additionally, by directly engaging with our patient population and making them active participants in their care, we participate in a growing movement toward patient-centered care and shared decision-making. We have demonstrated that the majority of patients can be taught to complete our app independently and that patients are enthusiastic about partnering with their providers in novel ways to optimize their recovery. Though the majority of participants had little difficulty completing the app, formal usability testing allowed us to identify components needing further improvement, providing invaluable information we could not have otherwise obtained. This argues strongly for the use of formal usability testing in the development of future novel protocols for patient-centered care.

## Abbreviations

AKA: above the knee amputation
BLE: bilateral lower extremity
SSI: surgical site infection
SUS: system usability scale
